# Paternal genetic affinity between western Austronesians and Daic populations

**DOI:** 10.1186/1471-2148-8-146

**Published:** 2008-05-15

**Authors:** Hui Li, Bo Wen, Shu-Juo Chen, Bing Su, Patcharin Pramoonjago, Yangfan Liu, Shangling Pan, Zhendong Qin, Wenhong Liu, Xu Cheng, Ningning Yang, Xin Li, Dinhbinh Tran, Daru Lu, Mu-Tsu Hsu, Ranjan Deka, Sangkot Marzuki, Chia-Chen Tan, Li Jin

**Affiliations:** 1 MOE Key Laboratory of Contemporary Anthropology and Center for Evolutionary Biology, School of Life Sciences and Institutes for Biomedical Sciences, Fudan University, Shanghai 200433, China; 2 Department of Genetics, School of Medicine, Yale University, New Haven CT 06520, USA; 3 Graduate Institute of Anthropology, Tzu Chi University, Hualien 970, Taiwan, China; 4 Department of Anthropological Sciences, Stanford University, Stanford, CA 94305, USA; 5 Key Laboratory of Cellular and Molecular Evolution, Kunming Institute of Zoology, Chinese Academy of Sciences, Kunming 650223, China; 6 Eijkman Institute for Molecular Biology, Jakarta, Indonesia; 7 Department of Pathophysiology, Guangxi Medical University, Nanning 530021, China; 8 Huê Medical College, Huê, Viêtnam; 9 Center for Genome Information, Department of Environmental Health, University of Cincinnati, Cincinnati, OH 45267, USA; 10 CAS-MPG Partner Institute for Computational Biology, SIBS, CAS, Shanghai 200013, China

## Abstract

**Background:**

Austronesian is a linguistic family spread in most areas of the Southeast Asia, the Pacific Ocean, and the Indian Ocean. Based on their linguistic similarity, this linguistic family included Malayo-Polynesians and Taiwan aborigines. The linguistic similarity also led to the controversial hypothesis that Taiwan is the homeland of all the Malayo-Polynesians, a hypothesis that has been debated by ethnologists, linguists, archaeologists, and geneticists. It is well accepted that the Eastern Austronesians (Micronesians and Polynesians) derived from the Western Austronesians (Island Southeast Asians and Taiwanese), and that the Daic populations on the mainland are supposed to be the headstream of all the Austronesian populations.

**Results:**

In this report, we studied 20 SNPs and 7 STRs in the non-recombining region of the 1,509 Y chromosomes from 30 China Daic populations, 23 Indonesian and Vietnam Malayo-Polynesian populations, and 11 Taiwan aboriginal populations. These three groups show many resemblances in paternal lineages. Admixture analyses demonstrated that the Daic populations are hardly influenced by Han Chinese genetically, and that they make up the largest proportion of Indonesians. Most of the population samples contain a high frequency of haplogroup O1a-M119, which is nearly absent in other ethnic families. The STR network of haplogroup O1a* illustrated that Indonesian lineages did not derive from Taiwan aborigines as linguistic studies suggest, but from Daic populations.

**Conclusion:**

We show that, in contrast to the Taiwan homeland hypothesis, the Island Southeast Asians do not have a Taiwan origin based on their paternal lineages. Furthermore, we show that both Taiwan aborigines and Indonesians likely derived from the Daic populations based on their paternal lineages. These two populations seem to have evolved independently of each other. Our results indicate that a super-phylum, which includes Taiwan aborigines, Daic, and Malayo-Polynesians, is genetically educible.

## Background

Austronesian is one of the most important linguistic families, spread in most regions of Island Southeast Asia, the Pacific Ocean, and the Indian Ocean, and comprising more than one fifth of all the languages in the world [1]. This linguistic family was originally proposed by Murdock [2] by bringing two groups of speakers, i.e. Malayo-Polynesians (Island Southeast Asians (ISEA), Malagasy, Micronesians, and Polynesians) and Taiwan aborigines together as a monophyletic unit based on their linguistic similarity [3,4]. Later, Benedict found that another linguistic family in East Asia, Daic, has many resemblances with the so-called Austronesian, and therefore announced a super-phylum of Austro-Tai [5]. Daic is a linguistic family located to the north of the ISEA groups, mainly in South China. Some Daic populations spread to Laos, Thailand, and as far as India [1]. Substantial resemblances among Taiwan aborigines, Malayo-Polynesians, and Daic speakers have been reported by ethnologists [6-10] and linguists [11-15], linking Taiwan aborigines and Malayo-Polynesians to coastal populations in Southeast China, primarily Daic speakers and their ancestry, *Baiyue*.

The origin of Austronesian has always been a controversial subject in linguistics and other related fields. The Express Train Hypothesis, a well accepted linguistic theory on the origin of Austronesian [3,4,16,17], postulates that proto-Austronesians originated in Taiwan and began to expand southward about 5,000–6,000 years ago by way of the Philippines and Eastern Indonesia. They eventually navigated eastward to Micronesia and Polynesia, and westward to Western Indonesia and Madagascar. The 'express train' refers to a rapid dispersal across the present Austronesian range starting from Eastern Indonesia. The hypothesis of the Taiwan origin of all the Austronesians (Taiwan Homeland Hypothesis or THH hereafter) is primarily based on the observation that a much higher linguistic diversity exists among languages of Taiwan aborigines than among the Malayo-Polynesians [3,4]. However, some linguists found evidences against the THH, and suggested that Kalimantan or Sulawesi may be the homeland of Austronesian [15,18,19]. The THH was further challenged by ethnologists [6-9], archaeologists [10], and geneticists [20-25].

Genetic evidence has been equally controversial. Some mitochondrial DNA (mtDNA) studies suggested a Taiwan origin of Polynesians [20-22]. A recent mtDNA study on Taiwan aborigines found a root of the "Polynesian Motif" in Taiwan, which suggests that the THH may be confirmed in maternal lineages [26]. On the other hand, this theory was challenged in paternal lineages by the Y-Chromosome studies that showed a lack of resemblance between the Polynesians and Taiwan aborigines [23]. It was also challenged by other mtDNA studies, which suggest an Indonesian origin of Polynesians [24,25]. The conflicts in the genetic evidence can be attributed to the lack of evidence or populations from two crucial regions: (1) coastal populations in Southeast Asia ancestral to three Austronesian groups (Taiwan aborigines, ISEA, and Polynesians), and (2) ISEA populations including Indonesians from which Polynesians derived.

**Figure 1:**
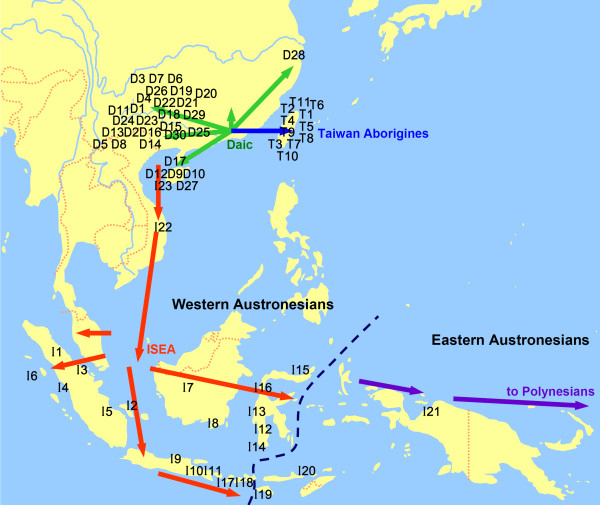
**Geographic distribution of sampled populations and migration routes suggested by Y chromosome analysis**. The codes for the population samples are the same as those in Table 1. Green arrows indicate expansion of Daic; blue arrows, Taiwanese; orange arrows, ISEA. The origin of Polynesians, purple arrows, remains controversial in paternal lineages.

Another important factor in the genetic structure of Austronesians is that Eastern Austronesians are distinctly different from Western Austronesians (ISEA and Taiwan aborigines, Figure 1). Autosomal STR variation studies [27] revealed a pronounced genetic division between Polynesians and Western Austronesians. These studies suggest that the Polynesians might have undergone natural selection or have been admixed with Melanesians. This process changed their genetic structure [16,20,28]. There is also the possibility of genetic drift and founder effects during the dispersal of Polynesians. The genetic structure of Western Austronesians, especially that of the ISEA, is more pivotal to the origin of Austronesians (Figure 1). The high Y chromosome diversity of Indonesian populations, Bali and Sumba islanders, suggests that these populations have existed since the Palaeolithic age [29,30]. Because of this high genetic diversity, it appears that the ISEA, especially the Indonesians are not just of Taiwanese origin.

Here, we examined the THH of ISEA by studying the Y chromosome diversity of all relevant population groups such as that of the Daic, Indonesians, and Taiwan aborigines. We show that the paternal lineages of both ISEA and Taiwan aborigines derived from the Daic, although independently of each other. In addition, our findings indicate that it is unlikely that Taiwan is the homeland of the paternal lineages of the ISEA populations.

## Results and Discussion

To determine the genetic affinity between the Daic populations and the Western Austronesians, we typed twenty single nucleotide polymorphisms (SNPs) and seven short tandem repeats (STRs) in the non-recombining region of 1,509 Y chromosomes sampled from 30 Daic populations, 23 ISEA populations, and 11 Taiwan aboriginal populations (see Figure 1 for locations of the populations and Table 1 for population information). Almost all of the Daic populations in China and all of the Taiwan aboriginal populations were sampled in this study.

**Figure 2:**
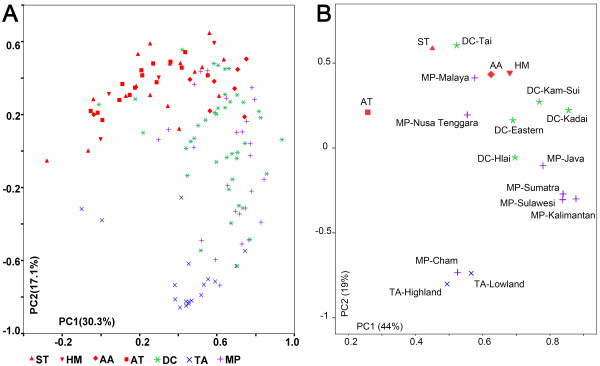
**Principal component plot of Y-SNP**. **(A) PC plot of all the population samples**. DC (green stars) is closest to MP (purple crosses) and TA (blue crosses). All of the other groups including ST, HM, AA, and AT (red spots including triangles, squares and diamonds) are rather far removed from MP and TA, which indicates that DC is the only group that might be related to MP and TA. **(B) PC plots of pooled samples**. The ST, HM, AA, and AT samples were pooled according to the linguistic families. The DC samples were pooled according to the sub-families. MP and TA samples were pooled according to the geographic locations. Ethnic groups: AA, Austro-Asiatic speakers; AT, Altaic speakers; DC, Daic speakers; HM, Hmong-Mien speakers; MP, Malayo-Polynesian speakers; ST, Sino-Tibetan speakers; TA, Taiwan aborigines.

In addition, principal component (PC) analysis of 134 East Asian populations encompassing all linguistic groups in East and Southeast Asia was performed using the frequencies of haplogroups defined by SNPs. The result showed that Daic populations are closer to the Western Austronesian groups than any other East and Southeast Asian populations are (Figure 2), indicating a strong genetic affinity between Daic speakers and Western Austronesians. The separation of the Daic-ISEA-Taiwan cluster from the other ethnic groups is attributable to PC2 rather than to PC1, and O1a* is the haplogroup that shows the strongest correlation with PC2 (*r*^2 ^= -0.875, *P *< 10^-4^; see Additional file 1 for details). Furthermore, O1a-M119 is the dominating haplogroup in Taiwan aborigines (average 77%) ranging from 54% to 100% (Table 2, sum of O1a* and O1a2). This lineage is also highly prevalent in Daic speakers (20.5%) and in ISEA (21.2%), but not in the other East Asians (< 5%) [23,31-34]. Therefore, O1a-M119 is expected to provide much information for delineating the relationship between the Daic and Western Austronesians.

The PC plot of Figure 2 indicates that some Daic populations are close to the Sino-Tibetan cluster. It is possible that Daic and Sino-Tibetan populations have a common ancestry, which might have resulted in their genetic resemblance. However, another explanation for this observation is that Daic populations in mainland East Asia may have been influenced by Han Chinese genetically as they coexisted as neighbors since around 2,500 years ago. Admixture analysis can estimate the proportions of assumed Daic or Han ancestry in the present Daic populations, and some Daic populations isolated from Han Chinese can be used as the parental population in this admixture analysis. Aboriginal populations on Hainan Island (Hlai, Jiamao, and Cun) and Taiwan Island are assumed to have been relatively isolated, as their cultures were little influenced by the exotic cultures on the mainland. Therefore, the genetic structures of these island aborigines might be the closest to that of ancestral Daic [35].

**Table 1:** Classification, population, and location information of the populations sampled in this study

No.	ETHNIC	ISO639-3	FAMILY	SUB-FAMILY	BRANCH	POPULATION	COUNTRY	PROVINCE	COUNTY
D1	Bolyu	ply	Austro-Asiatic	Mon-Khmer	Palyu	10,000	China	Guangxi	Longlin
D2	Yerong	yrn	Daic	Kadai	Bu-Rong	400	China	Guangxi	Napo
D3	Qau	gio	Daic	Kadai	Ge-Chi	3,000	China	Guizhou	Bijie
D4	Blue-Gelao	giq	Daic	Kadai	Ge-Chi	1,700	China	Guangxi	Longlin
D5	Lachi	lbt	Daic	Kadai	Ge-Chi	9,016	China	Yunnan	Maguan
D6	Mollao		Daic	Kadai	Ge-Chi	30,000	China	Guizhou	Majiang
D7	Red-Gelao	gir	Daic	Kadai	Ge-Chi	1,500	China	Guizhou	Dafang
D8	White-Gelao	giw	Daic	Kadai	Ge-Chi	1,200	China	Yunnan	Malipo
D9	Hlai-Qi	lic	Daic	Kadai	Hlai	747,000	China	Hainan	Tongza
D10	Jiamao	jio	Daic	Kadai	Hlai	52,300	China	Hainan	Baoting
D11	Buyang	byu	Daic	Kadai	Yang-Biao	3,000	China	Yunnan	Guangnan
D12	Cun	cuq	Daic	Kadai	Yang-Biao	70,000	China	Hainan	Dongfang
D13	Laqua	laq	Daic	Kadai	Yang-Biao	307	China	Yunnan	Malipo
D14	Man-Caolan	mlc	Daic	Kam-Tai	Be-Tai	114,000	China	Guangxi	Fangcheng
D15	Zhuang-N	ccx	Daic	Kam-Tai	Be-Tai	10,000,000	China	Guangxi	Wuming
D16	Zhuang-S	ccy	Daic	Kam-Tai	Be-Tai	4,000,000	China	Guangxi	Chongzuo
D17	Lingao	onb	Daic	Kam-Tai	Be-Tai	520,000	China	Hainan	Lingao
D18	E	eee	Daic	Kam-Tai	Be-Tai	30,000	China	Guangxi	Rongshui
D19	Ai-Cham	aih	Daic	Kam-Tai	Kam-Sui	2,300	China	Guizhou	Libo
D20	Dong/Kam	doc	Daic	Kam-Tai	Kam-Sui	907,560	China	Guangxi	Sanjiang
D21	Sui	swi	Daic	Kam-Tai	Kam-Sui	345,993	China	Guangxi	Rongshui
D22	Mak	mkg	Daic	Kam-Tai	Kam-Sui	10,000	China	Guizhou	Libo
D23	Mulam	mlm	Daic	Kam-Tai	Kam-Sui	159,328	China	Guangxi	Luocheng
D24	Maonan	mmd	Daic	Kam-Tai	Kam-Sui	37,000	China	Guangxi	Huanjiang
D25	Biao	byk	Daic	Kam-Tai	Kam-Sui	20,000	China	Guangdong	Huaiji
D26	Then	tct	Daic	Kam-Tai	Kam-Sui	20,000	China	Guizhou	Pingtang
D27	Danga		Daic	Unclassified		1,000,000	China	Hainan	Lingshui
D28	DornQdayc		Daic	Unclassified		500,000	China	Shanghai	Minhang
D29	CaoMiao	cov	Daic	Kam-Tai	Kam-Sui	63,632	China	Guangxi	Rongshui
D30	Laka	lbc	Daic	Kam-Tai	Kam-Sui	12,000	China	Guangxi	Jinxiu
T1	Amis	ami	Austronesian	Taiwan	Paiwanic	130,000	China	Taiwan	Hualien
T2	Pazeh	uun	Austronesian	Taiwan	Paiwanic	300	China	Taiwan	Cholan
T3	Siraiya-Makatao	fos	Austronesian	Taiwan	Paiwanic	10,000	China	Taiwan	Hualien
T4	Thao	ssf	Austronesian	Taiwan	Paiwanic	248	China	Taiwan	Nantou
T5	Paiwan	pwn	Austronesian	Taiwan	Paiwanic	53,000	China	Taiwan	Taitung
T6	Atayal	tay	Austronesian	Taiwan	Atayalic	63,000	China	Taiwan	Yilan
T7	Rukai	dru	Austronesian	Taiwan	Paiwanic	8,007	China	Taiwan	Pingtung
T8	Pyuma	pyu	Austronesian	Taiwan	Paiwanic	8,132	China	Taiwan	Taitung
T9	Tsou	tsu	Austronesian	Taiwan	Tsouic	5,797	China	Taiwan	Kagi
T10	Bunun	bnn	Austronesian	Taiwan	Paiwanic	34,000	China	Taiwan	Hualien
T11	Saisiyat	xsy	Austronesian	Taiwan	Paiwanic	4,194	China	Taiwan	Yilan
I1	Batak	bbc	Austronesian	Malayo-Polynesian	Western	5,800,000	Indonesia	Sumatera Utara	
I2	Bangka	mly	Austronesian	Malayo-Polynesian	Western	500,000	Indonesia	Sumatera Selatan	Bangka
I3	Malay (Riau)	mly	Austronesian	Malayo-Polynesian	Western	2,000,000	Indonesia	Riau	
I4	Minangkabau	min	Austronesian	Malayo-Polynesian	Western	4,000,000	Indonesia	Sumatera Barat	
I5	Palembang	plm	Austronesian	Malayo-Polynesian	Western	1,100,000	Indonesia	Sumatera Selatan	
I6	Nias	nia	Austronesian	Malayo-Polynesian	Western	600,000	Indonesia	Sumatera Utara	Nias
I7	Dayak	dyk	Austronesian	Malayo-Polynesian	Western	2,100,000	Indonesia	Kalimantan Tengah	
I8	Banjar	bjn	Austronesian	Malayo-Polynesian	Western	3,000,000	Indonesia	Kalimantan Selatan	
I9	Javanese	jav	Austronesian	Malayo-Polynesian	Western	75,500,000	Indonesia	Jawa Tengah	
I10	Tengger	tes	Austronesian	Malayo-Polynesian	Western	500,000	Indonesia	Jawa Timur	
I11	Balinese	ban	Austronesian	Malayo-Polynesian	Western	3,800,000	Indonesia	Bali	
I12	Bugis	bug	Austronesian	Malayo-Polynesian	Western	3,500,000	Indonesia	Sulawesi Selatan	
I13	Toraja	sda	Austronesian	Malayo-Polynesian	Western	500,000	Indonesia	Sulawesi Selatan	
I14	Makasar	mak	Austronesian	Malayo-Polynesian	Western	1,600,000	Indonesia	Sulawesi Selatan	
I15	Minahasa	tom	Austronesian	Malayo-Polynesian	Western	200,000	Indonesia	Sulawesi Utara	
I16	Kaili	lew	Austronesian	Malayo-Polynesian	Western	471,000	Indonesia	Sulawesi Tengah	
I17	Sasak	sas	Austronesian	Malayo-Polynesian	Western	2,100,000	Indonesia	Nusa Tenggara Barat	Lombok
I18	Sumbawa	smw	Austronesian	Malayo-Polynesian	Western	400,000	Indonesia	Nusa Tenggara Barat	Sumbawa
I19	Sumba	xbr	Austronesian	Malayo-Polynesian	Central	234,574	Indonesia	Nusa Tenggara Timur	Sumba
I20	Alor	aol	Austronesian	Malayo-Polynesian	Central	25,000	Indonesia	Nusa Tenggara Timur	Alor
I21	Irian		Geelvink Bay			20,806	Indonesia	Irian Jaya	
I22	Cham	cjm	Austronesian	Malayo-Polynesian	Western	99,000	Vietnam	Binhdinh	
I23	Tsat	huq	Austronesian	Malayo-Polynesian	Western	4,500	China	Hainan	Sanya

Detailed information can be searched online http://www.ethnologue.com by ISO639-3 codes.

**Table 2:** Y-SNP haplogroup frequencies of the newly studied samples (%)

Population	Size	C	D*	D1	F	M	K	O*	O1a*	O1a2	O2a*	O2a1	O3*	O3a1	O3a4	O3a5	O3a5a	P
Bolyu	30		3.3				3.3	10.0	10.0	3.3	23.3		30.0			6.7	10.0	
Yerong	16										62.5	6.3	18.8			12.5		
Qau	13		15.4				7.7	23.1			15.4		30.8				7.7	
Blue Gelao	30						3.3	13.3	60.0		16.7		3.3			3.3		
Lachi	30	3.3		3.3	13.3		13.3	16.7	6.7		10.0		3.3			6.7	23.3	
Mollao	30	10.0					3.3	13.3	3.3	3.3	63.3		3.3					
Red Gelao	31	3.2					6.5	22.6	22.6		16.1		12.9				16.1	
White Gelao	14							35.7	14.3		42.9					7.1		
Hlai-Qi	34							35.3	32.4		29.4						2.9	
Jiamao	27							25.9	51.9		22.2							
Buyang	32		3.1		6.3		6.3	9.4	3.1		71.9							
Cun	31	3.2					6.5	9.7	38.7				38.7				3.2	
Laqua	25							32.0	4.0		60.0					4.0		
Man-Caolan	30	10.0					10.0	53.3	3.3		20.0					3.3		
Zhuang-N	22							13.6		4.6	72.7			4.6			4.6	
Zhuang-S	15							13.3	20.0		60.0	6.7						
Lingao	30						3.3	16.7	26.7		13.3		3.3			10.0	26.7	
E	31	3.2			3.2		9.7	16.1	6.5		54.8		3.2			3.2		
Laka	23	4.4	52.2				4.4				8.7		26.1		4.4			
Kam/Dong	38	21.1					5.3	10.5			39.5		10.5			2.6	10.5	
Sui	50				8.0		10.0		18.0		44.0					20.0		
Mak&AiCham	40						2.5				87.5		5.0			2.5		2.5
Mulam	40	2.5		12.5	7.5		5.0		5.0	25.0	30.0		7.5			5.0		
Maonan	32	9.4			9.4		15.6				56.3		9.4					
Biao	34	2.9						5.9	14.7		17.7		52.9					5.9
Then	30		3.3					3.3	33.3		50.0						6.7	3.3
Danga	40	20.0	5.0		2.5		7.5	17.5	7.5	5.0	17.5					2.5	15.0	
DornQdayc-S	74	2.1		6.3					39.6	12.5	8.3		4.2			27.1		
DornQdayc-N	51	5.9	2.0				2.0	31.4	29.4		2.0		2.0			11.8	13.7	
CaoMiao	33						8.2		10.0		3.0		66.7			12.1		
Amis	28							7.1	42.8	17.8	7.1		21.4			3.6		
Pazeh	21						14.3		38.1	19.1	14.3		14.3					
Makatao	37	2.7					2.7	5.4	70.3		5.4					13.5		
Thao	22						4.6		81.8	4.6			9.1					
Paiwan	22								63.6	27.3						9.1		
Atayal	22								95.5				4.5					
Rukai	11								81.8	18.2								
Pyuma	11								72.7	9.1			9.1					9.1
Tsou	18								88.9	5.6			5.6					
Bunun	17						5.9		17.6	58.8		17.6						
Saisiyat	11								45.5	9.1	9.1	9.1	27.3					
Batak	13						11.6	19.3	23.1		15.4		23.1					7.7
Bangka	13	7.7					7.7		30.8		23.1		23.1		7.7			
Malay	13				7.7		7.7	7.7	38.5		7.7		23.1					7.7
Minangkabau	15				6.7		20.0	20.0			13.3		20.0					20.0
Palembang	11	9.1							63.6		18.2		9.1					
Nias	12											8.3	91.7					
Dayak	15				6.7		26.7		20.0	20.0	6.7	6.7	13.3					
Banjar	15	13.3			6.7				26.7		26.7		26.7					
Javanese	15						26.7	26.7	20.0		13.3		13.3					
Tengger	12	16.7					8.3		33.3		33.3				8.3			
Balinese	14						28.6	14.3	7.1		28.6		14.3		7.1			
Bugis	15				13.3		20.0		33.3				26.7				6.7	
Torajan	15				13.3		13.3	13.3	13.3	6.7	33.3				6.7			
Minahasa	14					7.1	50.0		21.4		7.1		14.3					
Makassar	13	23.1							30.8	15.4	7.7		23.1					
Kaili	15	6.7					33.3		20.0		6.7		26.7					6.7
Sasak	15	13.3					13.3	26.7	6.7		20.0		20.0					
Sumbawa	18						16.7						83.3					
Sumba	14				14.3		78.6						7.1					
Alor	13	38.5					30.7						23.1					7.7
Irian	11	45.5				36.4	18.2											
Cham	11							9.1	90.9									
Tsat	31	12.9						16.1	58.1		3.2					6.5	3.2	

Zhuang and DornQdayc are divided into Southern (S) and Northern (N) parts.

To estimate the assumed genetic influence of Han Chinese on the mainland Daic, we applied the Y SNP data of mainland Daic, Hainan aborigines, Taiwan aborigines, and Han Chinese [34] to our admixture analysis. For this analysis, we set the latter three pooled populations as the parental populations of mainland Daic. Our results show that the genetic contribution of the Hainan aborigines is very high (2.145 ± 0.927), while those of the Han Chinese (-0.314 ± 0.422) and Taiwan aborigines (-0.831 ± 0.662) are hardly detected. Here the negative values of the genetic contribution estimated by the ADMIX program suggest that there is no possible contributions to the present Daic populations. This result indicates that the paternal lineages of Daic populations are relatively undisturbed, and the genetic affinity between Daic and Western Austronesian populations has hardly been influenced by population admixture.

The ISEA populations may also be admixed. In our study, we assumed that the ISEA were mixed by three potential parental populations: Daic populations, Taiwan aborigines, and the indigenous populations of the Sunda Islands, who are similar to Papuans. We performed an admixture analysis on the Indonesians, and included data of the Papuans from the literature [36,37] as one of the parental population structures in the analysis. Our analysis showed the following admixture proportions: Daic (0.713 ± 0.124), Taiwan (0.143 ± 0.125), and Papuans (0.144 ± 0.050), indicating that the contribution of the Daic ancestry on the Indonesians is the most dominant. There is some uncertainty in these data as our assumption that the ISEA population is an admixture can not be tested.

As the haplogroup O1a* is the most unique haplogroup of the Daic and Western Austronesian populations, we estimated pairwise genetic divergence between Daic, Indonesians, and Taiwan aborigines using seven STRs carried by O1a* individuals (see Table 3 for genetic distances and Additional file 2 for STR raw data). Our study shows that the divergence between Taiwan aborigines and Indonesians is the largest, and is about 3-fold as much as that between the Daic group and Taiwan aborigines. The divergence between the Daic group and Indonesians is comparable to that between the Daic group and Taiwan aborigines. These findings indicate that the Indonesians and Taiwan aborigines are genetically closer to the Daic group than the two Western Austronesian groups are to each other. Furthermore, the diversity based on the seven STRs carried by O1a* individuals is higher in the Daic speakers than the diversities in Indonesians and Taiwan aborigines (Table 3). The population with the highest diversity is not always the oldest, but can also be a result of admixture with other neighbouring populations. However, the high diversity of the O1a* haplogroup of the Daic speakers should have resulted from the oldest age of the population, as this haplogroup is almost absent in the neighbouring populations and no admixture can bring more diversity. Taking the results of diversity and divergence together, the Daic population group is likely the ancestral group from which the Indonesians and Taiwan aborigines derived separately in paternal lineages. Other haplogroups of Y chromosomes (e.g. O3-M122, O2a-M95) displayed a similar pattern as O1a*, showing that the Daic group is genetically closer to Indonesians and Taiwan aborigines than these latter two groups are to each other (Table 3). Interestingly, O2a may be traced even further to Austro-Asiatic populations as suggested by a recent study [38].

**Table 3:** Y-STR diversity of O1a, O2a, and O3 haplogroup

Between-group Diversity (Genetic distance)									
	R_ST_			Linearized R_ST_					
	O1a	O2a	O3	O1a	O2a	O3			

Daic-TA	0.109 (p < 10^-5^)	0.012 (P = 0.271)	0.019 (P = 0.187)	0.122	0.012	0.019			
Daic-ISEA	0.108 (p < 10^-5^)	0.093 (P < 10^-5^)	0.049 (P = 0.001)	0.121	0.102	0.052			
TA-ISEA	0.269 (p < 10^-5^)	0.318 (P < 10^-5^)	0.285 (P < 10^-5^)	0.368	0.466	0.398			

Within-group Diversity									

	Size			Average Gene Diversity			Average Variance		
	O1a	O2a	O3	O1a	O2a	O3	O1a	O2a	O3

Daic	140	292	145	0.601	0.518	0.658	0.938	1.041	1.494
ISEA	75	38	64	0.547	0.397	0.498	0.897	0.320	0.634
TA	147	12	14	0.503	0.543	0.621	0.656	0.685	1.220

Y STR genetic distances between Taiwan Aborigines (TA) and ISEA were always largest and more than twice as much as those between Daic and one of the TA and ISEA groups among the samples of all of the three haplogroups, O1a, O2a and O3. Two statistics of R_st _and Linearized R_st _referred to reference [53].

Y STR within-group gene diversities of Daic were always largest. Average gene diversity refers to reference [55], and variance refers to reference [56].

**Figure 3:**
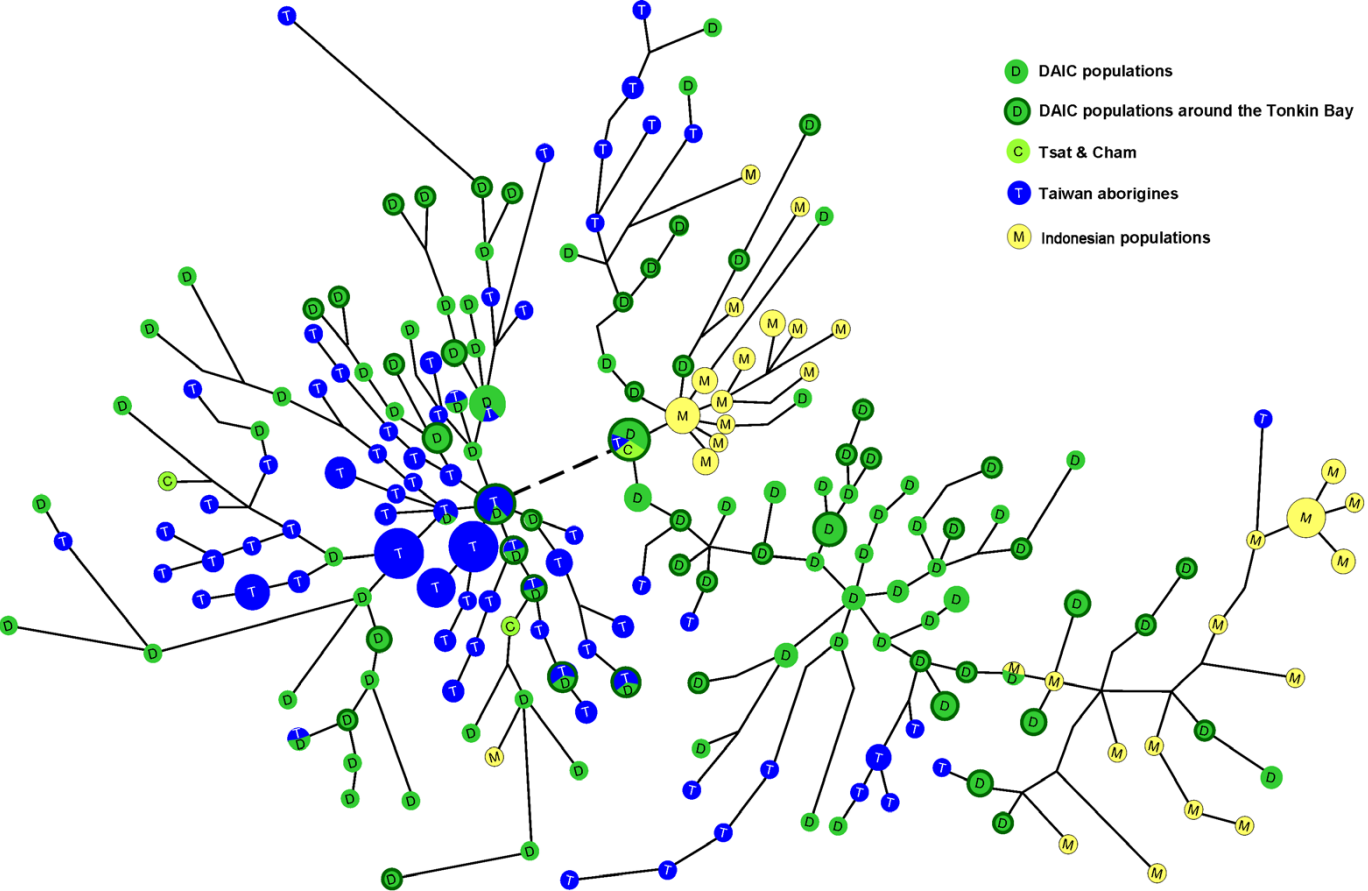
Haplotype network of Y-STRs of Haplogroup O1a* individuals. As the original network was too complicated to display, here we presented the shortest tree of the largest possibility reduced from the network (this function is available in the recent versions of NETWORK program). Each node represents an O1a* STR haplotype. The lengths of the lines are proportional to the mutation steps. The broken line stands for only one step. The sizes of the nodes are proportional to their frequencies. Almost none of the ISEA haplotypes is directly linked to Taiwan aborigines, and both ISEA and Taiwanese are linked directly or indirectly to the Daic haplotypes holding the centre of the network (big green node).

A median-joining network was constructed based on 7-STR haplotypes of O1a* individuals in the three ethnic groups (Figure 3). If THH of ISEA is true, i.e., ISEA primarily derived from Taiwan aborigines, one would expect sharing and/or connections of ISEA lineages and Taiwan aboriginal lineages in the network. In Figure 3, Daic lineages (green nodes) constitute the center of the network. All ISEA lineages (yellow nodes) and Taiwan aboriginal lineages (blue nodes) are either shared or connected to one of the Daic lineages, either directly or indirectly. In contrast, none of the Taiwan aboriginal lineages (except for one) are shared with or connected to the ISEA lineages. These observations suggest that ISEA did not directly derive from Taiwan aborigines but that the ISEA and Taiwan aborigines derived from the Daic independently of each other.

We further noticed the Daic lineages that are connected to ISEA lineages in the network. Interestingly, most of the Daic haplotypes connecting to the ISEA are either from Hainan Island or from Guangxi, which is to northwest of Hainan (green nodes with dark green frames in Figure 3). These Hainan and Guangxi populations are located around the Gulf of Tonkin. In particular, Cham, a Malayo-Polynesian population in South Vietnam, as well as Tsat in Hainan, which is a subgroup of Cham [11,39], were found to connect Daic and Indonesians in the network. Therefore, we hypothesized that the ISEA likely originated in the area around the Gulf of Tonkin, and migrated southward through the Indochina Peninsula to the Malaya Peninsula before they spread to most of the islands of the Pacific Ocean and the Indian Ocean.

The age of the O1a* haplogroup was estimated in the network. The total age is 33765 ± 5221 years, which corresponds to the last Ice Age. The age of all the Daic samples in the network is 33193 ± 5577 years, close to the age of O1a*. It is not easy to estimate the real age of the Taiwan clusters as they overlap with the Daic haplotypes to a large extent. This kind of overlap also indicates multiple migrations from Daic populations to Taiwan aborigines. We estimated the age of the Taiwan cluster in the left side of the network to be 14659 ± 3110 years. The estimated age of all the Taiwan samples is 21268 ± 3148 years. Interestingly, this latter age is close to the age of the oldest human remains found in Taiwan, those of the *Chochen *Man [40]. Therefore, we conclude that the migration of O1a* individuals from the mainland to Taiwan Island occurred during the Palaeolithic Age.

Because two fairly specific clusters of ISEA haplotypes can be observed in the network, we performed time estimates in both clusters. The age of the left ISEA cluster in the network is 9895 ± 2393 years, whereas that of the right cluster is 25880 ± 7137 years. The linguistic estimate for the origin of the Malayo-Polynesian is younger than that of our estimates, around 5000–6000 years ago [16]. Moreover, little overlap between Daic haplotypes and ISEA haplotypes is observed in the network, which indicates bottleneck effects might have formed the two ISEA clusters during the emigration of ISEA populations out of the ancestral Daic populations. Geographically, the bottleneck might be the narrow seashore of Vietnam. Therefore, the O1a* haplogroup was most probably introduced into ISEA populations during the origin of the Malayo-Polynesians more than 7500 years ago. However, the possibility of recent migrations of the O1a individuals into ISEA can not be ignored, because the genetic time estimate is not precise enough to eliminate such a possibility.

It should be noted that, in the Express Train Hypothesis, there are two different aspects: 1) the origin of the migrations, i.e. the Taiwan Homeland Hypothesis, and 2) the mode of migrations, i.e., a rapid dispersal starting from Indonesia. In this study, we examined the THH in Western Austronesians by including the Daic speakers and ISEA, both of which are largely missing in previous studies. We show that Taiwan is not likely the homeland of Indonesian ISEA, at least not for the major paternal lineages. Although both Taiwan aborigines and Indonesian ISEA derived from the Daic, their departures occurred separately, suggesting that the major paternal lineages of Western Austronesian populations are not monophyletic.

Interestingly, the spread of the domestic pig in the Southeast Asia archipelago and the Pacific took place in almost the same way as that of Western Austronesian populations suggested by our study. The pigs in Taiwan and in regions as far as Micronesia came directly from the mainland of East Asia, while those in the Southeast Asian archipelago and Polynesia came from the Indochina Peninsula. It is assumed that the domestic pig was introduced by human populations during early migrations, which would imply that humans have also entered the Southeast Asia archipelago and the Pacific in two different routes [41].

In fact, our observations are consistent with a monophyletic Austro-Tai super-phylum which contains Daic speakers, Malayo-Polynesians, and Taiwan aborigines [5]. The observations presented in this study demonstrate that it is absolutely necessary to include Daic populations and ISEA in the Austronesian origin studies. Without these groups, Polynesians and Taiwan aborigines would have appeared most similar to each other, leading to the conclusion that all the Austronesians originated in Taiwan.

Our results suggest that the Gulf of Tonkin is more likely the homeland of the paternal lineages of ISEA. Due to the complex nature of population migrations from Eastern Indonesia to the Pacific Islands [23,42-47], and the pronounced genetic division between Eastern and Western Austronesians [27], we opted not to include Polynesian data in our analysis. Instead, we only analyzed Western Austronesians. The absence of O1a-M119 in Polynesian populations is intriguing and it can not be simply explained by invoking the bottleneck effect [21-25] given that a great deal of diversity of Y chromosome haplotypes has been observed in Polynesians [23,42].

Consistent with our findings for paternal lineages, mitochondrial DNA studies on populations from Peninsular Malaysia also suggest an ancestry of aboriginal Malays in Indochina around the time of the Last Glacial Maximum [48]. This ancestry subsequently dispersed through the Malaya Peninsula into island Southeast Asia [48]. The ISEA mtDNA studies also indicated that if an Austronesian migration from Taiwan did take place, it was demographically minor [49].

Most of our conclusions are based on the analysis of O1a*, which is only a fraction of the Y-chromosome lineages found in these populations. The frequency of this group of lineages is remarkable in Taiwanese populations, but it is not so dramatic in Malayo-Polynesians or Daic populations. It is possible that some population events could have involved other Y-chromosome lineages. It is also reasonable that there are other minor parts of paternal lineages with different origins, such as aboriginal populations of Indonesia prior to the formation of Austronesian, or that more recent migrations from South Asia took place [29]. The genetic relationship amongst the East and Southeast Asians are much more complicated than expected.

## Conclusion

Our results show that the Daic populations are closer to the Western Austronesian populations in paternal lineages than any other ethnic groups in East Asia are. The STR diversity of the Y chromosome haplogroup O1a-M119, the major haplogroup among the Daic and Western Austronesian populations, shows that Taiwan and ISEA, two groups of Western Austronesian, derived from the Daic independently of each other. Therefore, it is most likely that the ISEA populations mainly originated in the region around the Tonkin Gulf, the homeland of the Daic, and migrated to Indonesia through the Vietnam corridor. In contrast, the Taiwan aborigines migrated from mainland China directly. Our results indicate that a super-phylum, which includes Taiwan aborigines, Daic, and Malayo-Polynesians, is genetically educible.

## Methods

### Sampling

Blood samples from 30 Daic populations across South China were collected using FTA cards (Whatman^® ^Inc), covering almost all of the Daic populations in China. Those from 11 Taiwan aborigine populations were collected from both the lowlands and the highlands of Taiwan. Samples from 23 Malayo-Polynesian populations were collected, among which 21 were collected across Indonesia, 1 from Binhdinh of Vietnam, and 1 from Hainan of China. The sample sizes from each population are given in Table 2. All of the 1,509 individuals studied from these populations are unrelated and gave their consents for this study. Individual samples were from diverse regions of the population distribution area to make the sample more diverse. Reference data for 70 other groups in East and Southeast Asia were obtained from the literature (including some Daic speaking populations [23], Malayo-Polynesians [23], Taiwan aborigines [23], Tibeto-Burman speaking populations [31-33], Han Chinese [31,34], and Altaic speaking populations [31]), for a total reference sample size of 1,348 individuals. In PC analysis, these samples refer to a total of 134 different population groups, including newly typed and previously published populations.

Although the sample sizes of some populations were relatively small, we do not think it is necessary to enlarge these sample sizes, as they were collected from very small populations with low Y chromosome diversity, such as the Ai-Cham and Geelvink Irians. The effective population size of the Y chromosome is usually less than one fourth of the size of that of autosomes. Therefore, Y chromosome diversity studies require much smaller sample sizes than studies of autosomal genetic markers. For a normal size population of some hundred thousand, a sample of around 30 individuals will be sufficient. Even fewer samples are required for small populations. Here we maintained a sample size of around 30 for most of the populations, and around 15 for small populations.

### Genetic markers

Twenty bi-allelic Y-chromosome markers (SNP), YAP, M15, M130, M89, M9, M5, M122, M134, M7, M117, M121, M111, M17, M175, M119, M110, M95, M88, M45, and M120 were typed by PCR-based restriction-fragment length polymorphism methods [31]. Most of these markers are highly informative in East Asians and define 19 haplogroups following the Y Chromosome Consortium nomenclature [50].

Seven microsatellite markers (STR) on Y-chromosome, DYS19, DYS388, DYS389-1, DYS390, DYS391, DYS392, and DYS393 were typed using fluorescent-labelled primers [51]. The genotyping results are given in Additional file 2.

### Data analysis

Population relationships were investigated with principal component analyses using Y-chromosome haplogroup frequencies and SPSS11.0 software (SPSS Inc.). Some of the SNPs, such as M175 and M117, were not typed for the previously published populations, therefore our O*-M175 data were combined into haplogroup K, and O3a5a-M117 into O3a5* in our PC analysis. Correlation analysis among haplogroups and PCs was also conducted using SPSS11.0.

The admixture analysis was performed using an ADMIX 2.0 program [52] in order to evaluate the genetic influence of Han Chinese on the Daic populations. We assumed the potential admixture started 2,500 ago when the Qin army entered the Daic area in Canton. The admixture proportions of the Indonesians were also estimated by ADMIX 2.0, and the admixture history was to start 5,000 years ago.

The genetic distances among Daic, Taiwan aborigines, and Malayo-Polynesians were estimated by R_ST _and linearized R_ST _[53] using ARLEQUIN software [54], and the diversities of three groups were evaluated by average gene diversity, haplotype diversity [55], and variance of the STR allele sizes [56].

A Median-Joining network of O1a* STR haplogroups was drawn by Network 4.1 software (Fluxus Technology Ltd). The age of O1a* was estimated in the network. The mutation rate used in the time estimate is 1.932 × 10^-4 ^per year, the sum of the mutation rates [57] of all the STRs used in the network. We assumed 25 years for one generation.

## Authors' contributions

HL, SJC, BS, YL, PP, ZQ, WL, XC, XL, and NY carried out the molecular genetic studies. HL and LJ drafted the manuscript. HL, BW, DL, MH, RD, SM, CCT, and LJ participated in the design of the study and performed the statistical analysis. HL, SJC, PP, SP, and DT collected the samples. All authors read and approved the final manuscript.

## Supplementary Material

Additional file 1 Correlation coefficients between haplogroups and PCs. Although P values of the correlation coefficients between PC1 and M9, M110, M95, M88 etc. are all very significant, all of these correlation coefficients are less than 0.5. Thus, PC1 has little information about the ethnic clustering. In contrast, PC2 is significantly correlated with O1a-M119 seen in a large correlation coefficient. This haplogroup distinguishes the Daic-MP-TW cluster. Thus, PC2 provides information on ethnic clustering. Click here for file

Additional file 2 Y-STR haplotypes of individual samples. The names of the individuals begin with ISO639-3 codes of their populations. Click here for file
