# Supplementary material for: Paternal genetic affinity between western Austronesians and Daic populations
Source: BMC Evol Biol. 2008 May 15;8:146. doi: 10.1186/1471-2148-8-146 (PMC2408594; doi:10.1186/1471-2148-8-146)
Supplement: Additional file 1 — Correlation coefficients between haplogroups and PCs. Although P values of the correlation coefficients between PC1 and M9, M110, M95, M88 etc. are all very significant, all of these correlation coefficients are less than 0.5. Thus, PC1 has little information about the ethnic clustering. In contrast, PC2 is significantly correlated with O1a-M119 seen in a large correlation coefficient. This haplogroup distinguishes the Daic-MP-TW cluster. Thus, PC2 provides information on ethnic clustering. [file 1471-2148-8-146-S1.doc]

**Additional file 1 -** Correlation coefficients between haplogroups and PCs

|  | M130 | YAP | M15 | M89 | M9 | M122 | M7 | M134 | M119 | M110 | M95 | M88 | M120 | M45 | M17 | M5 |
| --- | --- | --- | --- | --- | --- | --- | --- | --- | --- | --- | --- | --- | --- | --- | --- | --- |
| PC1 | -0.141 | -0.214 | -0.238 | -0.057 | 0.475 | 0.079 | -0.121 | -0.205 | 0.080 | -0.373 | 0.336 | -0.385 | -0.277 | -0.198 | 0.072 | 0.084 |
| P | 0.112 | 0.015 | 0.007 | 0.524 | 0.000 | 0.375 | 0.172 | 0.020 | 0.365 | 0.000 | 0.000 | 0.000 | 0.001 | 0.025 | 0.419 | 0.342 |
| PC2 | 0.029 | 0.182 | 0.075 | 0.154 | 0.411 | 0.071 | 0.014 | 0.321 | **-0.875** | -0.288 | 0.302 | 0.040 | -0.109 | -0.015 | 0.064 | 0.021 |
| P | 0.747 | 0.039 | 0.399 | 0.082 | 0.000 | 0.423 | 0.871 | 0.000 | 0.000 | 0.001 | 0.001 | 0.653 | 0.221 | 0.865 | 0.472 | 0.814 |

Although P values of the correlation coefficients between PC1 and M9, M110, M95, M88 etc. are all very significant, all of these correlation coefficients are less than 0.5. Thus, PC1 has little information about the ethnic clustering.

In contrast, PC2 is significantly correlated with O1a-M119 seen in a large correlation coefficient. This haplogroup distinguishes the Daic-MP-TW cluster. Thus, PC2 provides information on ethnic clustering.
